# Autophagy suppresses the pathogenic immune response to dietary antigens in cystic fibrosis

**DOI:** 10.1038/s41419-019-1500-x

**Published:** 2019-03-15

**Authors:** Valeria R. Villella, Speranza Esposito, Eleonora Ferrari, Romina Monzani, Antonella Tosco, Federica Rossin, Alice Castaldo, Marco Silano, Gian Luigi Marseglia, Luigina Romani, Nikolai A. Barlev, Mauro Piacentini, Valeria Raia, Guido Kroemer, Luigi Maiuri

**Affiliations:** 1 European Institute for Research in Cystic Fibrosis, San Raffaele Scientific Institute, Milan, Italy; 2 Department of Health Sciences, University of Eastern Piedmont, Novara, 28100 Italy; 3 Regional Cystic Fibrosis Center, Pediatric Unit, Department of Translational Medical Sciences, Federico II University Naples, Naples, 80131 Italy; 4 Department of Biology, University of Rome “Tor Vergata”, Rome, Italy; 5 Department of Food Safety, Nutrition and Veterinary Public Health, Istituto Superiore di Sanità, Roma, Italy; 6 Dipartimento di Pediatria, Fondazione IRCCS Policlinico San Matteo, Pavia, Italy; 7 Department of Experimental Medicine, University of Perugia, Perugia, Italy; 8 Gene Expression Laboratory, Institute of Citology, Saint-Petersburg, Russia; 9 Equipe11 labellisée Ligue Nationale contrele Cancer, Centre de Recherche des Cordeliers, Paris, France; 10 INSERM U1138, Centre de Recherche des Cordeliers, Paris, France; 11 Université Paris Descartes, Paris, France; 12 Metabolomics and Cell Biology Platforms, Institut Gustave Roussy, Villejuif, France; 13 Pôle de Biologie, Hôpital Européen Georges Pompidou, AP-HP, Paris, France; 14 Suzhou Institute for Systems Biology, Chinese Academy of Sciences, Suzhou, China; 15 Karolinska Institute, Department of Women’s and Children’s Health, Karolinska University Hospital, Stockholm, 17176 Sweden

## Abstract

Under physiological conditions, a finely tuned system of cellular adaptation allows the intestinal mucosa to maintain the gut barrier function while avoiding excessive immune responses to non-self-antigens from dietary origin or from commensal microbes. This homeostatic function is compromised in cystic fibrosis (CF) due to loss-of-function mutations in the CF transmembrane conductance regulator (CFTR). Recently, we reported that mice bearing defective CFTR are abnormally susceptible to a celiac disease-like enteropathy, in thus far that oral challenge with the gluten derivative gliadin elicits an inflammatory response. However, the mechanisms through which CFTR malfunction drives such an exaggerated response to dietary protein remains elusive. Here we demonstrate that the proteostasis regulator/transglutaminase 2 (TGM2) inhibitor cysteamine restores reduced Beclin 1 (BECN1) protein levels in mice bearing cysteamine-rescuable F508del-CFTR mutant, either in homozygosis or in compound heterozygosis with a null allele, but not in knock-out CFTR mice. When cysteamine restored BECN1 expression, autophagy was increased and gliadin-induced inflammation was reduced. The beneficial effects of cysteamine on F508del-CFTR mice were lost when these mice were backcrossed into a *Becn1* haploinsufficient/autophagy-deficient background. Conversely, the transfection-enforced expression of *BECN1* in human intestinal epithelial Caco-2 cells mitigated the pro-inflammatory cellular stress response elicited by the gliadin-derived P31–43 peptide. In conclusion, our data provide the proof-of-concept that autophagy stimulation may mitigate the intestinal malfunction of CF patients.

## Introduction

Cystic fibrosis (CF) is the most frequent monogenic lethal disease affecting more than 85,000 subjects worldwide^1–4^. CF is caused by loss-of-function mutations in the gene coding for the cystic fibrosis transmembrane conductance regulator (CFTR)^5,6^, a protein with 1480 amino acid residues that belongs to the ABC transport family and functions as a cyclic AMP-regulated anion channel. CFTR is expressed in, and is relevant to the function of, many tissues, including airways, small and large intestine, pancreas, biliary tree, male reproductive tract and sweat glands^3,7^, but it is also expressed in central nervous system, leukocytes, smooth muscle and cartilage of the large airways^7^. Approximately 2000 mutations have been identified in the *CFTR* gene and are categorized in 6 classes according to their impact on the synthesis (class I), processing (class II), gating (class III), conductance (class IV), quantity (class V) and recycling (class VI) of the CFTR protein^8–11^. Among, these mutations, the clinically most important one is the F508del-CFTR mutation (class II), which accounts for 70–90% of CFTR cases.

CF is best known for its respiratory phenotype, as the abnormal anion transport results in increased mucin polymer cross-links and mucus viscosity^12–14^, leading to accumulation of thick, sticky mucus in the lung. These events cause chronic inflammation, persistent and untreatable bacterial colonization and recurrent chest infections, mostly by *Pseudomonas aeruginosa, Staphylococcus aureus*, and *Burkholderia cepacia*^15^. Chronic infection and inflammation ultimately lead to progressive lung disease with bronchiectasis and tissue destruction, culminating in respiratory insufficiency^15^. Defective CFTR function also frequently leads to intestinal problems^16,17^, including intestinal obstruction as well as an exaggerated immune response to dietary antigens^18–20^. Indeed, a constitutive inflammation at both airway and intestinal mucosa, is a feature of CF^17,19,20^. Moreover, CF patients often show serum antibodies against dietary antigens^18,20^.

Beyond its function as an ion channel^16^, CFTR orchestrates proteostasis at respiratory and intestinal epithelial surfaces, thus regulating adaptation to cell-autonomous or external stress^21–24^. CFTR malfunction causes a maladaptive epithelial stress response with increased generation of reactive oxygen species (ROS) which oxidize and activate tissue transglutaminase (TGM2)^25,26^. Activated TGM2 targets several substrates, among which Beclin 1 (BECN1), a major pro-autophagic protein that acts as an allosteric activator of phosphatidylinositol 3-kinase catalytic subunit type 3 (PIK3C3)^21–23^. Transamidation of BECN1 by TGM2 dislodges the BECN1 interactome away from the endoplasmic reticulum (ER), resulting in the functional sequestration of PIK3C3 into intracellular aggregates. This causes inhibition of autophagy, accumulation of the autophagic substrate sequestosome 1 (SQSTM1) and reduced availability of the PIK3C3 product phosphatidyl-inositol-3-phosphate (PtdIns3P) at early endosomes that impairs endosomal maturation and trafficking^21,22^. Mismanaged proteostasis in epithelial cells consequent to CFTR malfunction also leads to TGM2-mediated crosslinking of the anti-inflammatory peroxisome-proliferator-activated-receptor-γ (PPARγ), as well as increased nuclear translocation of nuclear factor kappa-light-chain-enhancer of activated B cells (NF-κB) owing to TGM2 targeting of NF-κB inhibitor alpha (NFKBIA) within histone-deacetylase 6 (HDAC6)^+^/vimentin^+^ intracellular aggresomes^21,23,25,26^. NF-κB activation results in increased levels of pro-inflammatory cytokines, among which interleukin (IL)-17A, IL-21 and IL-15, a master cytokine involved in gut homeostasis^18,23,27–29^ as well as IL-1β^18^.

Importantly, CFTR malfunction, TG2 activation and autophagy deficiency are engaged in a self-amplifying feed-forward loop. For this reason, inhibition of TGM2 by cysteamine is sufficient to restore autophagy and to favor the expression of functional CFTR at the epithelial surface. Indeed, treatment of neonatal mice bearing the F508del-CFTR mutation with cysteamine can prevent intestinal obstruction^30^. Moreover, cysteamine efficiently restores CFTR function and reduces lung inflammation in patients carrying at least one class II CFTR mutation^30,31^. A combination of two proteostasis regulators, cysteamine and the autophagy inducer epigallocatechin gallate (EGCG), was particularly efficient in restoring the expression and function of the mutant F508del-CFTR protein^11,31^.

CF patients exhibit a three-fold increase in the prevalence of celiac disease (CD)^18,32,33^ an extremely frequent permanent intolerance to gluten/gliadin proteins that occurs in a proportion of susceptible individuals bearing the human leukocyte antigen (HLA) DQ2/DQ8^34–36^. Accordingly, CFTR defective mice exhibit an increased susceptibility to the enteropathogenic effects of gliadin, a common dietary protein present in gluten from wheat, rye, and barley^18^. Gliadin inhibited the function of CFTR in human enterocytes and mouse models of CD through a direct molecular interaction involving a specific gliadin-derived peptide (P31–43) with the nucleotide-binding domain-1 (NBD1) of CFTR^18^. Indeed, the effects of gliadin on enterocyte proteostasis are reminiscent of those observed in CFTR defective mice. Given the pivotal role of BECN1 and autophagy in orchestrating proteostasis in CF epithelia, we investigated whether the increased responsiveness to gliadin in CF mice may be due to defective autophagy and whether re-establishing BECN1 levels and autophagy by means of cysteamine would protect the CF intestine against the detrimental effects of gliadin.

## Results

### Cysteamine restores CFTR function in *Cftr*^*F508del*^ mice after gliadin challenge

Cysteamine is reportedly effective in rescuing CFTR at the intestinal epithelial surface of mice homozygous for the *F508del-CFTR* mutation^30,31^. To investigate whether rescuing CFTR function by means of cysteamine would abrogate the pathogenic response to gliadin, we orally administered cysteamine for 5 consecutive days (60 µg/kg in 100 µl saline/day) to knock-in mice harboring the most common loss-of-function F508del-CFTR mutation (*Cftrtm1EUR*, F508del, FVB/129, *Cftr*^*F508del/F508del*^), CFTR knock-out mice (B6.129P2-KOCftrtm1UNC, *Cftr*^*−/−*^), knock-in mice harbouring one F508del-CFTR allele in combination with one null-CFTR allele (*Cftr*^*F508del/−*^) or their wild-type (WT) littermates (FVB/129 or B6.129P2). After or without cysteamine pre-treatment (60 µg/kg in 100 µl saline/day), mice were orally challenged with gliadin (5 mg/daily for 1 week and then 5 mg/daily thrice a week for 3 weeks) or vehicle for the following 4 weeks (*n* = 10 per group of treatment), according to established procedures^18^.

**Fig. 1:**
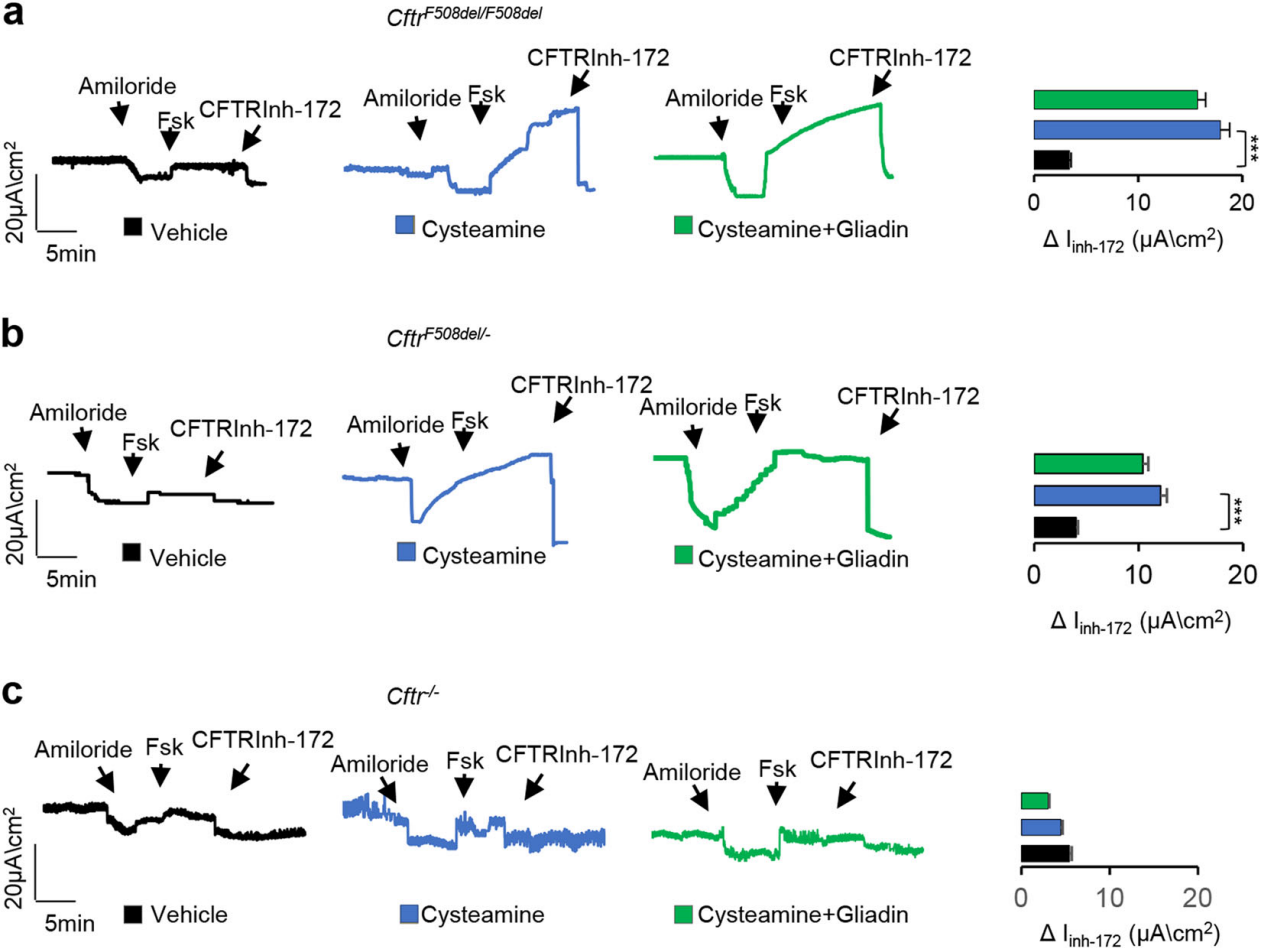
Cysteamine restores CFTR function in *Cftr*^*F508del*^ mice after gliadin challenge. **a** *Cftr*^*F508del/F508del*^ **b** *Cftr*^*F508del/*−^, and **c** *Cftr*^−*/*−^ mice orally treated with vehicle or cysteamine (60 µg/kg in 100 µl saline/day for 5 days) and then challenged with gliadin for consecutive 4 weeks (5 mg/daily for 1 week and then 5 mg/daily thrice a week for 3 weeks) in the presence or absence of cysteamine (60 µg/kg in 100 µl saline/day) (*n* = 10 mice per group of treatment). The CFTR-dependent Cl^−^ secretion was measured by forskolin-induced (Fsk) increase of the chloride current (Isc (μA/cm^2^) in small intestines mounted in Ussing chambers; quantification of the peak CFTR Inhibitor 172 (CFTRinh172)-sensitive Isc (∆Isc). ***p < 0.001 versus cysteamine (ANOVA, Bonferroni post hoc test)

Since gliadin is capable of impairing CFTR function in gliadin-sensitive CFTR-sufficient mice^18^, we first assessed whether the cysteamine-mediated rescue of F508del-CFTR function would persist after gliadin administration. To this aim, the small intestine from *Cftr*^*F508del/F508del*^ mice exposed to cysteamine and/or gliadin (*n* = 10 per group of treatment) were mounted in Ussing chambers and CFTR function was assessed as the forskolin (Fsk)-induced increase of chloride current (Isc (μA/cm^2^)). In *Cftr*^*F508del/F508del*^ and *Cftr*^*F508del/*−^ mice, the 5-day pre-treatment with cysteamine enhanced intestinal CFTR function, as expected^31^. This CFTR rescuing ability of cysteamine was not compromised by gliadin challenge (Fig. 1a, b). Of note, cysteamine restored CFTR function in gliadin-treated or vehicle-treated *Cftr*^*F508del/F508del*^ and *Cftr*^*F508del/−*^ mice, whereas it failed to do so in *Cftr*^*−/−*^ mice (Fig. 1c), in line with the idea that the positive effect of cysteamine requires the presence of ‘rescuable’ F508del-CFTR protein.

### Cysteamine protects *Cftr*^*F508del*^ mice from the effects of gliadin in vivo

**Fig. 2:**
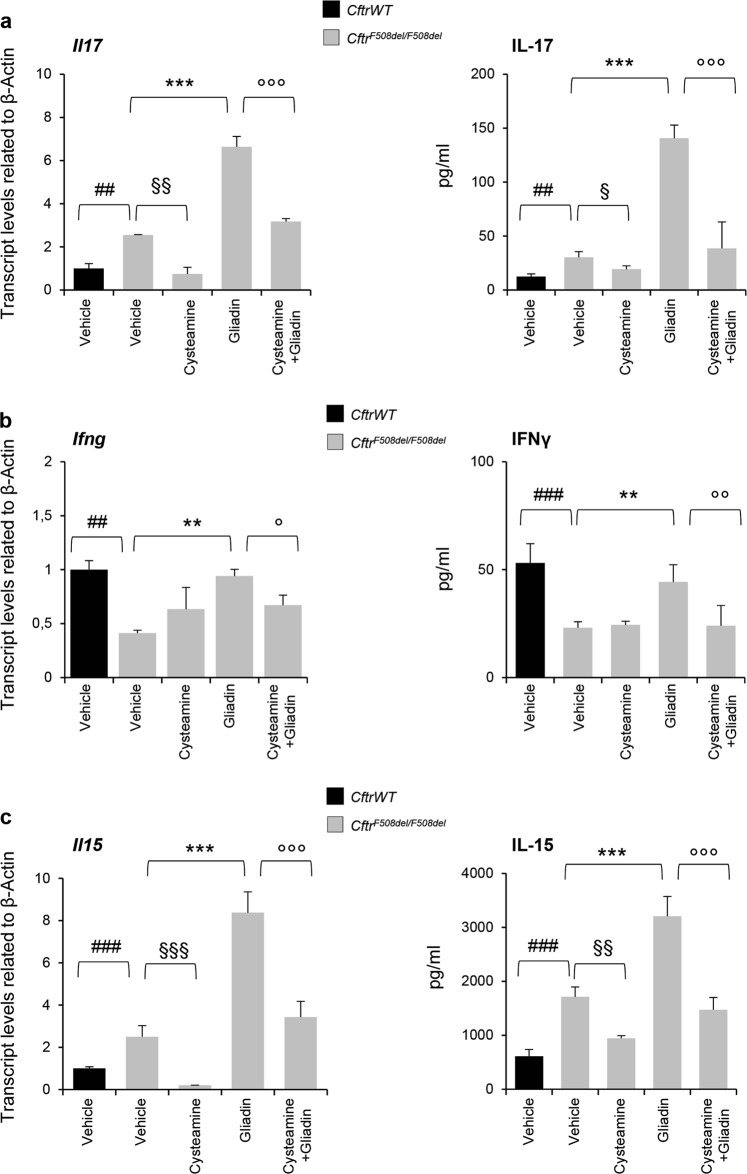
Cysteamine protects *Cftr*^*F508del*^ mice from the effects of gliadin in vivo. **a** IL-17A, **b** IFN-γ, and **c** IL-15 mRNA (*left*) and protein (*right*) levels in small intestine homogenates from *Cftr*^*F508del/F508del*^ or their *Cftr*^*WT*^ littermates treated with vehicle or cysteamine (60 µg/kg in 100 µl saline/day for 5 days) and then challenged with gliadin for consecutive 4 weeks (5 mg/daily for 1 week and then 5 mg/daily thrice a week for 3 weeks) in the presence or absence of cysteamine (60 µg/kg in 100 µl saline/day) (*n* = 10 per group). Means ± SD of pooled samples assayed in triplicates. ##*p* < 0.01 or ### *p* < 0.001 *Cftr*^*WT*^ versus *Cftr*^*F508del/F508del*^; §*p* < 0.05 or §§*p* < 0.01 or §§§*p* < 0.001 versus cysteamine treatment; **p < 0.01, ***p < 0.001 versus gliadin challenge; °*p* < 0.05 or °°*p* < 0.01 or °°°*p* < 0.001 versus cysteamine + gliadin (ANOVA, Bonferroni post hoc test)

Next, we investigated whether cysteamine would control the increased mucosal immune response that occurred in gliadin exposed *Cftr*^*F508del/F508del*^ mice. To this aim, we measured the levels of proinflammatory cytokines in small intestine homogenates from mice fed with gliadin for 4 weeks in the presence or absence of cysteamine. Cysteamine was effective in preventing the increased production of IL-17A and IFN-γ induced by gliadin (*p* < 0.01 and *p* < 0.001) (Fig. 2a, b). In addition, cysteamine controlled the production of IL-15, a master pro-inflammatory cytokine pivotal for driving the gliadin-induced enteropathy^37–41^ (Fig. 2c). Indeed, IL-15 is constitutively upregulated in mouse CF intestine and is significantly induced by oral gliadin challenge^18^. Again, the anti-inflammatory effect of cysteamine against gliadin-induced cytokine producton was observed in *Cftr*^*F508del/F508del*^, *Cftr*^*F508del/−*^ but not in *Cftr*^−*/−*^ mice (Supplementary Figure 1), supporting the hypothesis that cysteamine controls the gliadin-induced inflammation through restoring F508del-CFTR function.

### Cysteamine protects *Cftr*^*F508del*^ mice in vivo from the increased responsiveness to gliadin through restoring BECN1 and autophagy

**Fig. 3:**
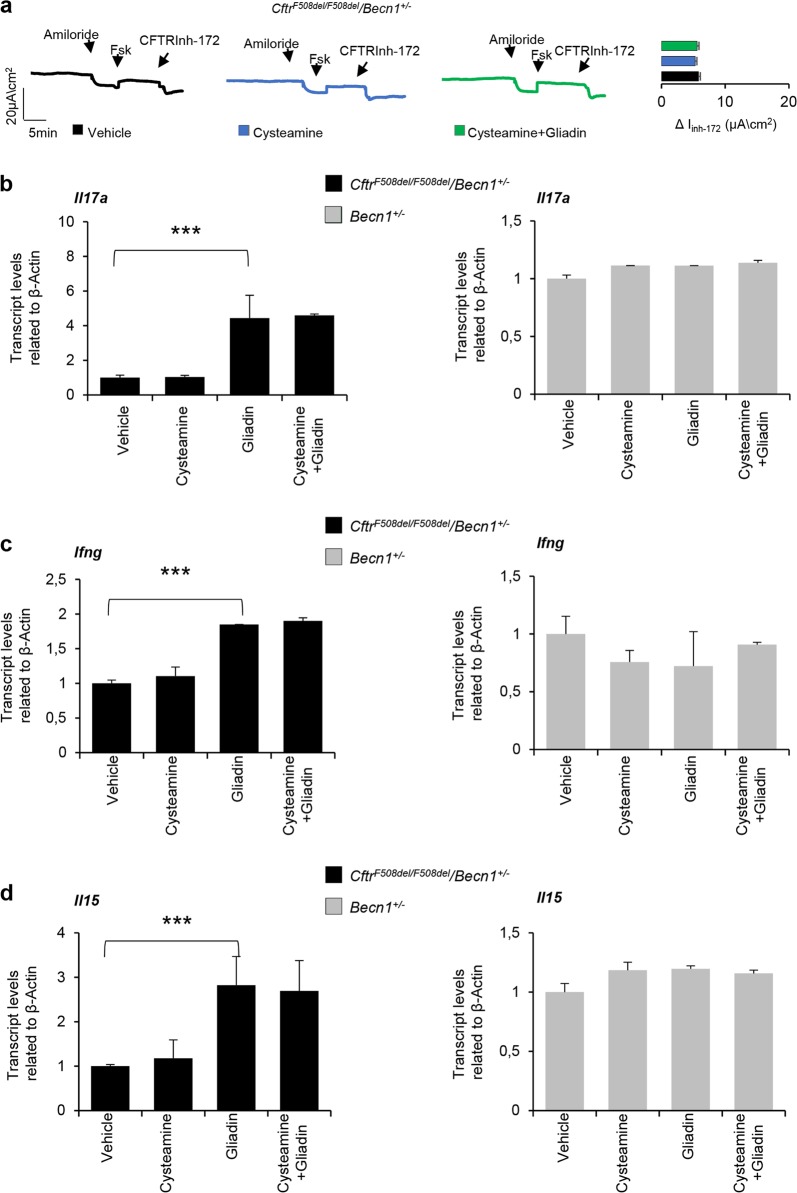
Cysteamine protects *Cftr*^*F508del*^ mice in vivo from the increased responsiveness to gliadin through restoring BECN1 and autophagy. **a** *Cftr*^*F508del/F508del*^*/Becn1*^*+/*−^ mice treated with cysteamine (60 µg/kg in 100 µl saline/day for 5 days) and then challenged with gliadin for consecutive 4 weeks (5 mg/daily for 1 week and then 5 mg/daily thrice a week for 3 weeks) in the presence or absence of cysteamine (60 µg/kg in 100 µl saline/day) (*n* = 10 mice per group of treatment). Assessment of CFTR-dependent Cl^−^ secretion measured by forskolin-induced (Fsk) increase of the chloride current (Isc (μA/cm^2^) in small intestines mounted in Ussing chambers; quantification of the peak CFTR Inhibitor 172 (CFTRinh172)-sensitive Isc (∆Isc). **b** IL-17A, **c** IFN-γ, and **d** IL-15 mRNA levels in small intestine homogenates from *Cftr*^*F508del/F508del*^*/Becn1*^*+/−*^ (left) or *Becn1*^+/−^ (right) mice treated with vehicle or cysteamine (60 µg/kg in 100 µl saline/day for 5 days) and then challenged with gliadin for consecutive 4 weeks (5 mg/daily for 1 week and then 5 mg/daily thrice a week for 3 weeks) in the presence or absence of cysteamine (60 µg/kg in 100 µl saline/day) (*n* = 10 per group of treatment). Means ± SD of pooled samples assayed in triplicates. ***p < 0.001 versus gliadin challenge; (ANOVA, Bonferroni post hoc test)

We previously reported that the inhibition of TGM2 with the subsequent restoration of BECN1 protein levels and autophagy are pivotal for allowing cysteamine to rescue F508del-CFTR at the epithelial surface^11,18,21,31^. For this reason, we investigated whether the protective effects of cysteamine against gliadin induced immune activation would be linked to its capacity to restore autophagy. To this aim, *Cftr*^*F508del/F508del*^ mice were backcrossed into a *Becn1* haploinsufficient background (to generate *Cftr*^*F508del/F508del*^*/Becn1*^*+/−*^ mice*)*^31^, and gliadin was orally administered upon optional pretreatment with cysteamine. Cysteamine was unable to restore the function of the intestinal CFTR (determined in Ussing chambers) from *Cftr*^*F508del/F508del*^*/Becn1*^*+/−*^ mice, either before or after gliadin challenge (Fig. 3a). Gliadin triggered an inflammatory response in *Cftr*^*F508del/F508del*^*/Becn1*^*+/−*^ mice, similarly to that observed in *Cftr*^*F508del/F508del*^ mice (Fig. 3b–d). Of note, cysteamine failed to mitigate the gliadin-elicited production of IL-15, IL-17A, and IFN-γ in *Cftr*^*F508del/F508del*^*/Becn1*^*+/−*^ mice (Fig. 3b–d). In conclusion, it appears that the haploinsufficiency of *Becn1* (genotype: *Becn1*^*+/−*^) abrogates the anti-inflammatory effects of cysteamine that is normally seen in *Cftr*^*F508del/F508del*^ mice.

### Restoring BECN1 protects intestinal epithelial cells from the detrimental effects of gliadin

**Fig. 4:**
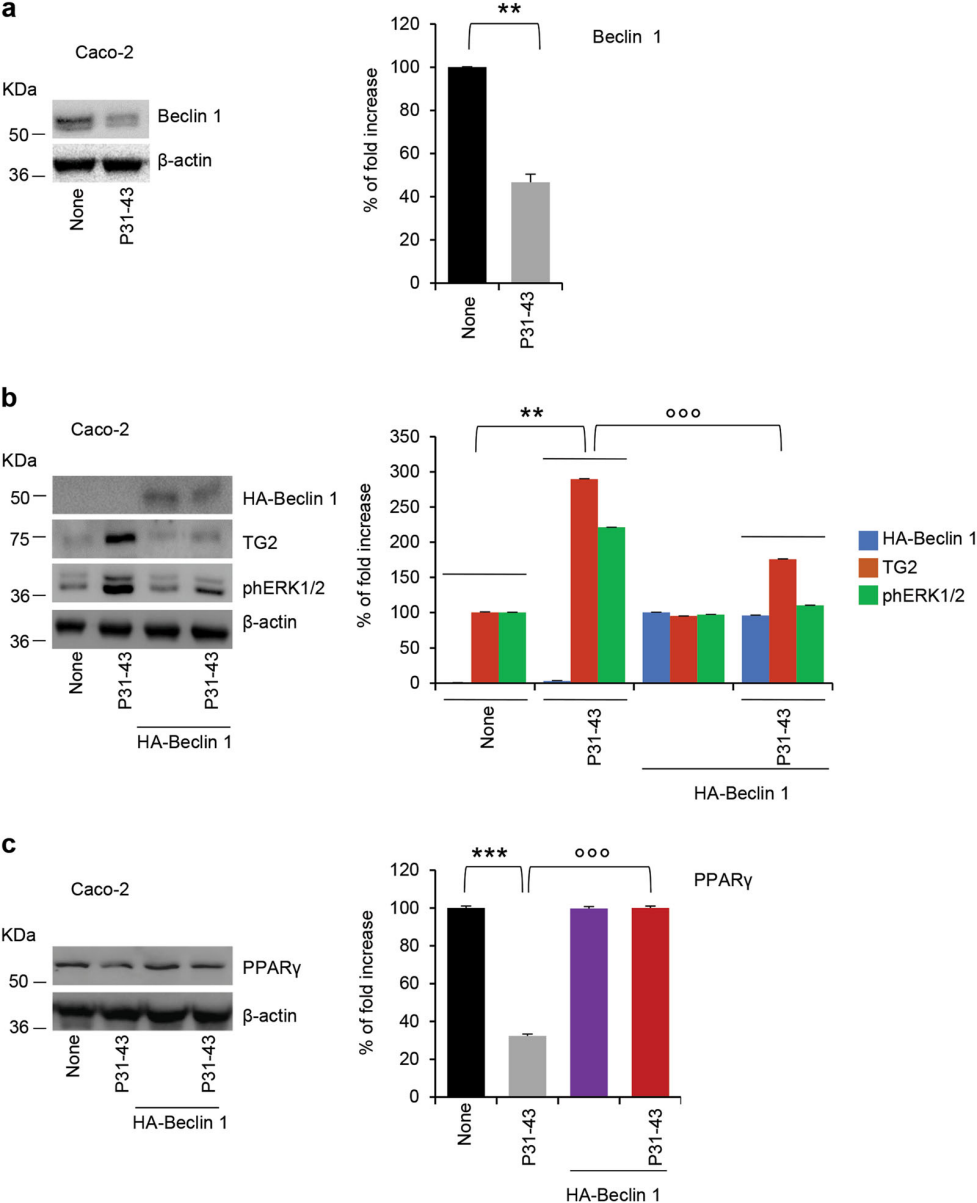
Restoring BECN1 protects intestinal epithelial cells from the detrimental effects of gliadin peptides. **a** Caco-2 cells treated with gliadin-derived P31–43 peptide or with vehicle for 3 h. Immunoblot with anti-Beclin 1 or anti-β-actin *(*left*)*, as loading control, in whole lysates and relative densitometric analysis (right) of immunoblot. Means ± SD of pooled samples assayed in triplicates; **p < 0.01 versus P31–43 (Student’s *t* test). **b**, **c** Caco-2 cells transfected with HA-Beclin 1 and then challenged for 3 h with P31–43. **b** Immunoblot of TGM2, phospho-ERK 1/2 (phERK 1/2) and with anti-HA tag for transfection control. Densitometric analysis of protein levels relative to β-actin (right). Means ± SD of pooled samples assayed in triplicates; **p < 0.01 versus P31–43, °°°*p* < 0.001 versus HA-Beclin1 + P31–43 (ANOVA, Bonferroni post hoc test). **c** Immunoblot of PPARγ *(*left*)* and densitometric analysis of protein levels relative to β-actin (right). Means ± SD of pooled samples assayed in triplicates; ***p < 0.001 versus P31–43, °°°*p* < 0.001 versus HA-Beclin1 + P31–43 (ANOVA, Bonferroni post hoc test)

To complete our demonstration that BECN1 and autophagy are crucial for the inflammatory effects of gliadin in intestinal epithelial cells, we resorted to human intestinal epithelial Caco-2 cells, which are reportedly sensitive to gliadin or gliadin-derived peptides^40^. When confluent Caco-2 cells were challenged for 3 h with a peptic-tryptic digest of gliadin from bread wheat (PT gliadin; 500 μg/ml)^40,41^ or the gliadin-derived peptide LGQQQPFPPQQPY (P31–43), CFTR function is inhibited^18^. Moreover, gliadin treatment of Caco-2 cells caused a reduction in BECN1 protein levels, as compared to unchallenged controls (Fig. 4a). Next, we transfected Caco-2 cells with HA-Beclin 1 and challenged them for 3 h with gliadin or P31–43. Of note, the enforced expression of BECN1 (which enhances the generation of autophagosomes and autophagy^21^), prevented signs of gliadin-induced inflammation, as it avoided the upregulation of TGM2, the activating phosphorylation of ERK 1/2 and the downregulation of PPARγ that were induced by gliadin (Fig. 4b, c). These results suggest that BECN1 plays an active role in mitigating the gliadin-induced inflammatory response of intestinal epithelial cells.

## Discussion

The proteostasis network is a system of cellular adaptation to endogenous or environmental stress. Mismanaged proteostasis contributes to a number of diseases arising as a result of inherited or stress-induced defects in protein conformation^42^. Autophagy is a major player of the proteostasis network as it regulates the turnover of large protein aggregates and even entire organelles. In addition, several components of the autophagy machinery dynamically interact with multiple signalling pathways to ensure intracellular homeostasis^43–45^. CF is the quintessential example of a disease characterized by major alterations of the proteostasis network^11,21,23,46^. Defective CFTR function highly compromises the capacity of cells to adequately respond to endogenous stress signals as well as to external challenges arising within the respiratory and gastrointestinal tracts^11^.

The intestine from CF patients is exposed to a particularly high antigenic load due to the frequent insufficiency of the exocrine pancreas^17^. Moreover, the local overactivation of the innate immune system compromises the handling of dietary molecules, thus favouring inadequate cellular and humoral immune responses to food components. Accordingly, CF patients often exhibit increased levels of antibodies against alimentary antigens, including anti-gliadin IgA antibodies, shifts in the intestinal microbiota, elevated fecal calprotectin levels and increased intestinal permeability^17,20,47^. Moreover, the prevalence of autoantibodies against TGM2 is four times higher than in the general population, even in the absence of histological evidence of intestinal lesions^18,32,33^. Indeed, when CFTR is disabled, the intracellular milieu undergoes major pathogenic changes. CFTR inhibition results in a ROS-mediated increase in the abundance and activity of TGM2^25,26^ with consequent functional sequestration of BECN1 complex and inhibition of autophagy^21–23^. Thus, CFTR inhibition, TGM2 activation and BECN1 inactivation act in concert to compromise proteostasis in the small intestine of CF mice, driving constitutive pro-inflammatory reactions that involves the activation of the NF-κB pathway and the NLRP3 inflammasome.

Here we demonstrate that functional BECN1 and autophagy are required to prevent the increased susceptibility of CF intestine to the gluten component gliadin. Indeed, the proteostasis regulator cysteamine was capable of reducing the pro-inflammatory effects of gliadin in mice bearing the most common F508del-CFTR mutant, either in homozygosity or in compound heterozygosity. Apparently, cysteamine abrogates the susceptibility of mice to oral gliadin challenge by acting as TGM2 inhibitor, thus preventing the BECN1 sequestration and autophagy impairement that normally result from CFTR inhibition. However, cysteamine fails to prevent gliadin-induced inflammation in CFTR KO mice, meaning that its effects are mediated by its ability to rescue CFTR function, as reported^23,31^. Importantly, the beneficial effects of cysteamine on F508del-CFTR mice are lost when these mice are backcrossed in an autophagy-deficient (*Becn1* haploinsufficient mice) background, indicating that the restoration of BECN1 levels and autophagy are indeed required to avoid the enteropathic effects of gliadin.

In aggregate, stimulating autophagy might represent a novel option to prevent intestinal manifestations of CF. In favor of this notion, it appears that enforced expression of BECN1 in gliadin-sensitive human intestinal epithelial cells^18,40^, effectively opposes the capability of the gliadin-derived P31–43 peptide^18,40,41^ to induce an epithelial stress response. In this perspective, and in line with the evidence that the best option to stimulate autophagy is to interfere with the function of its endogenous inhibitors^48^, it might be attempted to neutralize BECN1 inhibitory proteins. Druggable endogenous BECN1 antagonist include proteins from the BCL2 family (which can be targeted with so-called BH3 mimetics including ABT737, navitoclax, and venetoclax)^49^, the mechanistic target of rapamycin complex-1 (mTORC1) (which are inhibited by rapamycin, everolimus or tacrolimus)^48^, as well as the acetyltransferase EP300 (which is inhibited by aspirin, epigallocatechine gallate, or spermidine)^31,46,50,51^. Moreover, there is the option to directly inhibit TGM2 by cysteamine^31,52^ and to combine cysteamine with other autophagy stimulators such as EGCG^31^.

In conclusion, our data highlight the implication of CFTR in the suppression of diet-induced inflammation. CFTR may be viewed as a major stress sensor that alerts the autophagy machinery when a potentially harmful perturbation risks to perturb mucosal homeostasis. Once activated, autophagy then orchestrates the proper handling of luminal triggers by the intestinal mucosa. Pharmacological autophagy enhancement may be harnessed to prevent intestinal inflammation and to improve the nutritional status of CF patients.

## Materials and methods

### Cells and treatments

Human colon adenocarcinoma-derived Caco-2 were obtained from the ATCC. Cells were maintained in T25 flask in Modified Eagle Medium (MEM) supplemented with 10% fetal bovine serum (FBS), 2 mM Glutamine + 1% Non-Essential-Amino-Acids (NEAA) and the antibiotics penicillin/streptomycin (100 units/ml) (all reagents from Lonza)^18^. Cells were treated with 20 µg/ml of α-gliadin peptide P31–43 for 3 h synthesized by Inbios (Napoli, Italy). Cells were also treated with Pepsin-trypsin-gliadin (PT-gliadin) (500 μg/ml)^40,41,53^. Caco-2 cells were also transfected with HA-*beclin 1* and then treated with α-gliadin peptide P31–43 for 3 h.

### Plasmids and transfection

The pcDNA3-HA–*beclin 1* expression vector (a gift from N. Mizushima) was used for transfection experiments. Cells were transfected with pcDNA3-HA–*beclin 1* by means of Lipofectamine 2000 (Invitrogen) in accordance with the manufacturer’s instructions.

### Mice and treatments

CF mice homozygous for the F508del-CFTR in the FVB/129 outbred background (Cftrtm1EUR, F508del, FVB/129, abbreviated *Cftr*^*F508del/F508del*^) were obtained from Bob Scholte, Erasmus Medical CenterRotterdam, The Netherlands, CF coordinated action program EU FP6LSHMCT-2005-018932.50. Transgenic KO *Cftr* mice (B6.129P2-KOCftrtm1UNC, abbreviated *Cftr*^−/−^), were purchased from The Jackson Laboratory (Bar Harbor, ME, USA). The heterozygous *Cftr*^F508del/+^ males were backcrossed with heterozygous *Cftr*^*+/−*^ females to obtain F508del/null CFTR heterozygous mice (abbreviated *Cftr*^*F508del/−*^).

Female *Cftr*^*F508del/+*^ mice were backcrossed to the C57BL/6J background *Becn1*^*+/–*^ male mice (generous gift from Beth Levine, Center for Autophagy Research, Department of Internal Medicine, UT Southwestern Medical Center, Dallas, USA and Francesco Cecconi, University of Tor Vergata, Rome, Italy) to obtain at the first generation Becn1 haplo-insufficient F508del heterozygous mice (abbreviated *Cftr*^F508del/+^/*Becn1*^+/−^). These *Cftr*^*F508del/+*^*/Becn1*^*+/−*^ mice were crossbred to obtain Becn1 haplo-insufficient F508del homozygous mice (abbreviated *Cftr*^*F508del/F508del*^*/Becn1*^*+/−*^). The newly generated *Cftr*^*F508del/−*^ and the *Cftr*^*F508del/F508del*^*/Becn1*^*+/–*^ were housed at the San Raffaele Scientific Institute SOPF animal house (Milan, Italy).

Mice were challenged with vehicle alone or cysteamine (60 µg/kg in 100 µl saline/day) for 5 days. Mice were also challenged via gavage for 4 weeks with vehicle alone or gliadin (Sigma-Aldrich, G3375) (5 mg/daily for one week and then 5 mg/daily thrice a week for 3 weeks)^18^ in the presence or absence of cysteamine (60 µg/kg in 100 µl saline/day) challenge, (*n* = 10 mice per group of treatment).

Mice were anesthetized with Avertine (tribromoethanol, 250 mg/kg, Sigma Aldrich, Milan, Italy, T48402) and then killed and small intestines were collected. These studies and procedures were approved by the local Ethics Committee for Animal Welfare (IACUC No 849, 713) in compliance with European Community regulations for animal use in research (2010/63 UE).

### Ussing chamber

Chambers for mounting mouse tissue biopsies were obtained from Physiologic Instruments (model P2300, San Diego, CA, USA). Chamber solution was buffered by bubbling with identical Ringer solution on both sides and were maintained at 37 °C, vigorously stirred, and gassed with 95%O_2_/5%CO_2_. Tissues were short circuited using Ag/AgCl agar electrodes. A basolateral-to-apical chloride gradient was established by replacing NaCl with Na-gluconate in the apical (luminal) compartment to create a driving force for CFTR-dependent Cl^−^ secretion. To measure stimulated Isc, the changed sodium gluconate solution, after stabilization, was supplied with 100 µM amiloride. Agonists (forskolin) were added to the bathing solutions as indicated (for a minimum 5 min of observation under each condition) to activate CFTR channels present at the apical surface of the epithelium (either cell surface or lumen side of the tissue) and CFTR_Inh-172_ (10 µM) was added to the mucosal bathing solution to block CFTR-dependent Isc. Short-circuit current (expressed as Isc (μA/cm^2^)) and resistance were acquired or calculated using the VCC-600 transepithelial clamp from Physiologic Instruments and the Acquire &Analyze2∙3 software for data acquisition (Physiologic Instruments), as previously described^18,54^.

### Real-time and reverse transcription analysis

The analysis was performed as previously described^18,21,30,31,55^. Total RNA was extracted with the RNeasy Mini Kit (Qiagen, 74104) from mouse intestin homogenates. The mRNA was reverse transcribed with Onetranscript plus cDNA sintesis kit (abm good). The sequences of mouse primers were:^18^

IL-15: forward 5′-ACCAGCCTACAGGAGGCC-3′

reverse 5′- TGAGCAGCAGGTGGAGGTAC-3′

IL-17: forward 5′-ACCGCAATGAAGACCCTGAT-3′

reverse 5′- TCCCTCCGCATTGACACA-3′

INF-ϒ: forward5′-AGAGGATGGTTTGCATCTGGGTCA-3′

reverse5′- ACAACGCTATGCAGCTTGTTCGTG-3′

Expression levels of genes were normalized to β-actin (primer design HK-sy-mo600) levels in the same samples.

### Immunoblot

The whole lysate of cell lines and mice intestine homogenates were obtained from treated and untreated cells as described^21–26,55^. Equal amounts of protein were resolved by SDS-PAGE gel and blotted with antibodies against: PPARγ (Santa Cruz Biotechnology, sc7273) 1:500, BECN1 (Abcam, ab58878) 1:1000, HA (BD Bioscience) 1:5000, TG2 (CUB7402 Neomarker) 1:1000, phospho-ERK1/2 (phERK1/2, Cell Signaling Technology, #91101) 1:1000, Normalization was performed by probing the membrane with anti-β-actin (Cell Signaling, #4970) 1:1000.

### ELISA

ELISA analysis was performed on tissue samples using standard ELISA kits (R&D Systems) for IL-15, IL-17A, INF-γ, according to the manufacturer’s instructions. Samples were read in triplicate at 450 nm in a Microplate Reader (BioRad, Milan, Italy) using Microplate Manager 5.2.1 software. Values were normalized to protein concentration evaluated by Bradford analysis.

### Statistical analysis

GraphPad Prism software 6.01 (GraphPad Software) was used for analysis. Data are expressed as means ± SD. Statistical significance was calculated by ANOVA (Bonferroni’s post hoc test) for multiple comparisons and by Student’s *t*-test for single comparisons. We considered all *P* values 0.05 to be significant. The in vivo groups consisted of ten mice/group. The data reported are either representative of at least three experiments.

## Supplementary information

Supplementary Figure 1
