# Supplementary figures and images for: Autophagy suppresses the pathogenic immune response to dietary antigens in cystic fibrosis
Source: Cell Death Dis. 2019 Mar 15;10(4):258. doi: 10.1038/s41419-019-1500-x (PMC6420598; doi:10.1038/s41419-019-1500-x)

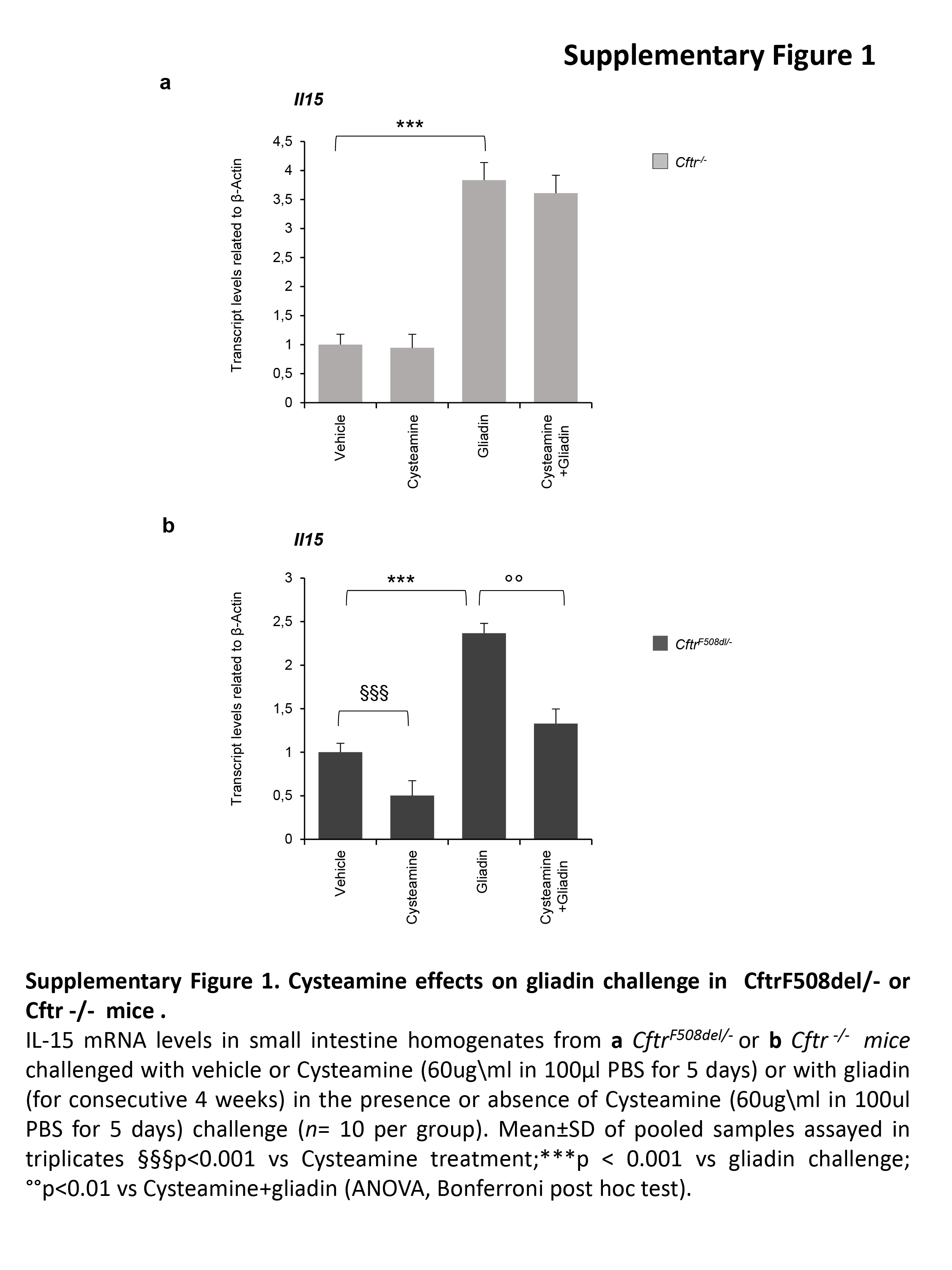

Supplement: Supplementary file 1 — Supplementary Figure 1 [file 41419_2019_1500_MOESM1_ESM.tif]
